# Mitigating Credit Risk through Corporate Governance: An Investigation into the Causal Pathways from Board Accountability to Reduced Non-Performing Loans in Uganda’s  Commercial Banks.

**DOI:** 10.12688/f1000research.171389.1

**Published:** 2025-10-14

**Authors:** Muniru Sewanyina, Michael Manyange, Tom Ongesa

**Affiliations:** 1 Business Administration, Kampala International University - Western Campus, Bushenyi, Western Region, Uganda; 2 Business Administration, Kampala International University - Western Campus, Bushenyi, Western Region, Uganda; 3 Business Administration, Kampala International University - Western Campus, Bushenyi, Western Region, Uganda

**Keywords:** Board Accountability, Non-performing Loans, Commercial Banks, Corporate Governance, Agency Theory

## Abstract

**Background:**

Non-performing loans (NPLs) pose a critical threat to the stability and financial performance of commercial banks globally. This study is grounded in Agency Theory, which highlights the risk of conflicts of interest between bank management (agents) and shareholders (principals). It investigates the specific role of board accountability as a governance mechanism in mitigating NPLs within the under-researched context of commercial banks in Western Uganda.

**Methods:**

A mixed-methods approach was employed. Quantitative data were collected via surveys from 195 bank employees and board members, selected through a combination of stratified, purposive, and simple random sampling from a population of 550, yielding an 84.1% response rate. The quantitative data were analyzed using descriptive statistics, Pearson correlation, and simple linear regression. Concurrently, qualitative data were gathered through interviews and analyzed using thematic analysis with NVivo software to provide depth and context.

**Results:**

The quantitative analysis revealed a statistically significant strong positive correlation between board accountability and the reduction of NPLs, confirming that heightened board oversight is associated with improved loan performance. The qualitative findings substantiate this, identifying two key mechanisms: first, reduced improper board interference in the loan approval process, and second, proactive board engagement in resolving existing NPLs. Together, the data triangulate to show that a board functioning with high integrity and clear accountability is pivotal in controlling NPLs.

**Conclusions:**

This study concludes that robust board accountability is a critical determinant of asset quality in commercial banks. It acts by aligning the interests of management and the board with those of shareholders, thereby curbing unauthorized influence and promoting prudent credit risk management. The findings underscore the importance of appointing high-integrity board members and strengthening governance structures. Future research should incorporate additional variables, such as macroeconomic conditions and regulatory frameworks, to develop a more comprehensive model of NPL determinants.

## Introduction

The prevalence of non-performing loans (NPLs) has led to financial instability in the banking sector, resulting in economic uncertainty. Sub-Saharan Africa has experienced a rise in the number of financial institutions failing due to inadequate corporate governance ( MacWilliam & Rafferty, 2017). Although corporate governance is a vital aspect of financial stability, research on its connection to NPLs is limited, particularly in Africa ( Nawaz, Nor, & Tolos, 2019). Board accountability in NPLs in Western Uganda is being examined to fill this gap.

The banking sector in Uganda has been plagued by several issues, including the closure of numerous banks due to the rise in NPLs. Among the institutions that were shut down by the Bank of Uganda were Teefe Bank (1993), International Credit Bank Ltd (1998), and Greenland Bank(1999). The National Bank of Commerce (2012) and Global Trust Bank (2014) have both faced comparable circumstances in recent times. Additionally, The rise in NPLs has caused a decline in available credit, with loan growth rates decreasing from 13.7% in 2011 to 3.2% in 2012, and then reaching 3.7% between 2015 and 2016. This research applies structural equation modeling (SEM) to the relationship between board accountability and NPLs, taking into account this situation.

## Review of related literature

The study is based on Agency Theory, which proposes that firm management acts as agents of action for shareholders, as proposed by Jensen and Meckling (1976). Breaches that default on their repayment obligations are known as NPLs and pose significant credit risk to banks. High NPL ratio, above 5% can be very detrimental to the stability of a bank ( Chariris, 2019). The influence of NPLs on corporate governance in Pakistan’s private, commercial, and state-owned banks was the subject of an inquiry by Ahmed et al. (2021). They found that board size is a strong predictor of NPLs, while board independence, ownership concentration, and government oversight are disadvantageous. However, their study did not address the central issue of board accountability in its investigation.

The presence of regulatory measures and attendance at board meetings is a significant factor in the loan loss provisions of Indian banks, as reported by Layola et al. (2016). According to Balagobei (2019), the impact of board activity on NPLs in Sri Lankan banks is not as significant as other factors like board size and CEO duality. Similarly, Tarchouna, Jarraya and Bouri (2022) established that smaller banks with weaker governance systems generally had higher NPLs, while midsize banks tend to have stronger governance structures. In spite of these insights, quantitative methods have not been employed in Ugandan studies before. The gap is addressed in this study using a mixed-methods approach, with the aim of further explaining the relationship between board accountability and NPLs in Uganda.

## Methodology

In this study, a mixed-methods approach is employed, with both cross–sectional and correlational focused research designs employed. 232 individuals, consisting of bank managers, board representatives, loan officers, and credit clients from three commercial banks in Western Uganda were surveyed for their data. Qualitative data was obtained through interviews with six purposively chosen participants, while quantitative data came from structured questionnaires.

SPSS version 24.0 employed descriptive statistics, correlations (for testing hypotheses) and regression analyses, all of which were conducted at a 5% significance level. The effects of board accountability on NPLs were studied through simulation using structural equation modeling (SEM) with Smart PLS 4.1.A.M. NVivo software was utilized to analyze qualitative data and then thematically focused on key themes and sub-themes.

## Results

### Qualitative findings

The quantitative investigation uncovered two primary concerns: (1) the degree of board meddling in loan transactions and (2) their involvement on the board’s part in managing issues within the NPL. Board members were noted to frequently use their influence on loan terms to benefit themselves or their colleagues, resulting in higher NPLs. The board’s efforts to improve loan recovery and bank performance were complemented by strategic steps such as supporting the credit department and implementing policies to reduce NPLs.

From Table 1, the findings also indicate that respondents agreed that banks disclose their corporate governance policies and guidelines as indicated by a high mean of 3.95 and confirmed by a low standard deviation of 0.901 in the same regard, the findings indicate that banks have separated chairman and CEO as indicated by high mean of 3.90 and confirmed by the low standard deviation of 0.87, also the findings show that all executive board members own shares after excluding options held as indicated by high mean of 3.90 and confirmed by the low standard deviation of 0.923. Findings also indicate that banks disclose a code of ethics for senior executives as shown by a high mean of 3.81 and confirmed by a low mean of 0.96, also the findings show that a board or a committee is responsible for CEO succession planning as shown by a high mean of 3.76 and confirmed by the standard deviation of 1.068. in the same regard, the findings indicate that members attended at least 75% of the board meetings as shown by a high mean of 3.71 and confirmed by a low standard deviation of 0.883.

**Table 1.** Descriptive statistics on board accountability.

Statements	N	Min.	Max.	Mean	Std. Deviation
Company discloses its corporate governance policies or guidelines	189	2	5	3.95	.901
Company has a separated chairman and CEO	189	2	5	3.90	.870
All executive board members own shares after excluding options held	189	2	5	3.90	.923
Company discloses a code of ethics for senior executives	189	2	5	3.81	.960
Board or a committee is responsible for CEO succession planning	189	1	5	3.76	1.068
All members attended at least 75% of the board meetings	189	2	5	3.71	.883
Company has failed to adopt the recommendations of a shareholder proposal	189	1	5	3.71	1.078
Non-executive board members have a formal session without executives once a year	189	1	5	3.67	1.042
Board members are subject to annual election by all shareholders	189	1	5	3.33	1.325
All non-executive board members own shares after excluding options held	189	2	5	3.14	.943
**Overall Mean and SD**	**189**			**3.69**	**0.999**

Primary data 2024.

Furthermore, the findings show that banks have failed to adopt the recommendations of a shareholder proposal as indicated by a high mean of 3.71 and confirmed by a standard deviation of 1.078, also the results indicate that non-executive board members have a formal session without executives once a year as shown by high mean of 3.67 and confirmed by the standard deviation of 1.042. The findings also indicate that board members are not subjected to annual election by all shareholders as shown by a moderate mean of 3.33 and supported by a standard deviation of 1.325 lastly, the respondents were neutral about that all non-executive board members own shares after excluding options held as shown by moderate mean of 3.14 and supported standard deviation of 0.943. the overall mean of 3.69 and standard deviation of 0.999 show that most respondents agreed with the statements that were used to measure board accountability.

### Correlation results

To examine the relationship between board accountability and non-performing loans of commercial banks, Pearson Linear Correlation Coefficient was used with the help of SPSS and the results were presented in Table 2. From Table 2, the results show that there is strong positive relationship between board accountability and non-performing loans of commercial banks (r=0.779, P=0.00<0.05). the relationship is statistically significant at 0.05, meaning that when members of the board are accountable by performing their duties following the guiding principles and polices, the non-performing loans of commercial banks reduces and the reverse is true.

**Table 2.** Correlation results on Board Accountability (BA) and Non-performing Loans (NP).

		NP	BA
NP	Pearson Correlation	1	.779 ^**^
	N	189	189
	Sig. (2-tailed)	.000	
BA	Pearson Correlation	.779 ^**^	1
	Sig. (2-tailed)	.000	
	N	189	189

** Correlation is significant at the 0.05 level (2-tailed).

### Regression results

From Table 3, the results show a strong positive overall relationship between board accountability and non-performing loans of commercial banks as indicated by R= 0.7749, and board accountability contributes 60.7% to non-performing loans of commercial banks as indicated by R ^2^= 0.607, meaning that when board members perform their roles very well, non-performing loans reduce by 60.7% and vice versa. The adjusted R square shows that a unit change in board accountability, causes 60.5% change in non-performing loans of commercial banks.

**Table 3.** Model summary.

Model	R	R Square	Adjusted R Square	Std. Error of the Estimate
1	.779 ^a^	.607	.605	.33141

^a^ Predictors: (Constant), BA.

From Table 4, degrees of Freedom (df ) indicate how many separate pieces of information are used to compute the sum of squares. In the case of the Regression, the df is 1, which is equal to the number of independent variables (predictors). The entire number of observations less the total number of predictors is the residual’s df, which is 187. Calculated by dividing the total of squares by the number of degrees of freedom, or mean square (MS). The residual MS is 0.110 and the regression MS is 31.722. F-value = 288.827 is the ratio of the Regression MS to the Residual MS. A greater F-value suggests that the model explains a considerable percentage of the variation. Sig.: This stands for the p-value, which is used to determine the statistical significance of the F-statistic. A score of.000 indicates that the predictors significantly explain the variation in the result, suggesting that the model is highly significant (p < 0.000). The dependent variable may be well explained by the independent variables (predictors) taken together, since the model has statistical significance (p-value =.000). With an F-value of 288.827, the model is able to explain a significant portion of the variation in comparison to the residual, or unexplained variance. Out of the total variation of 52.260, the model explains 31.722 of it, leaving residual variance of 20.538 unexplained.

**Table 4.** ANOVA ^a^.


Model		Sum of Squares	df	Mean Square	F	Sig.
1	Regression	31.722	1	31.722	288.827	.000 ^b^
	Residual	20.538	187	.110		
	Total	52.260	188			

^a^ Dependent Variable: NP.

^b^ Predictors: (Constant), BA.

From Table 5, without normalizing the units, they show the true effects of each independent variable (predictor) on the dependent variable. -The value of the dependent variable when all predictors are zero is called the intercept, and it is 2.917. Keeping all other factors equal, non-performing loans rise by 0.254 units for every unit increase in Board responsibility. The variable representing non-performing loans rises by 0.748 units for every unit increase in financial disclosure. These coefficients are obtained by standardizing the variables by placing them on a same scale, so that comparing the relative importance of each predictor is made simpler. Board accountability appears to have a moderately beneficial impact on non-performing loans (standardized beta = 0.689, t = 9.384, p = .000<0.05), this implies that null hypothesis (H0 _1_) which stated that there was no statistically significant relationship between board accountability and non-performing loans of commercial banks was rejected.

**Table 5.** Coefficients ^a^.

Model		Unstandardized Coefficients		Standardized Coefficients	t	Sig.
		B	Std. Error	Beta		
1	(Constant)	1.423	.152		9.384	.000
	BA	.689	.041	.779	16.995	.000

^a^ Dependent Variable: NP.

### Latent variable correlations

Latent variable correlations refer to the relationships between unobserved variables (latent variables) that are inferred from observed data. These correlations were typically estimated using statistical models such as structural equation modeling (SEM). The correlations represent the strength and direction of the linear relationship between pairs of latent variables as presented in Table 6.

**Table 6.** Latent correlation between board accountability and non-performing loans.

	Original sample (O)	Sample mean (M)	Standard deviation (STDEV)	T statistics (|O/STDEV|)	P values
BA <-> NP	0.904	0.912	0.022	40.658	0.000
BAb <-> NP	0.900	0.897	0.030	29.601	0.000

Primary data 2024.

From Table 6, the relationship between important latent variables, particularly Board Accountability, Oversight Risk Management (BA), Strategic Decision Making (BAb), and Non-Performing Loans (NP), is outlined in this table. To gauge the significance and strength of these correlations, one must refer to its first-ever sample correlation data, which includes standard deviations from previous measurements, t-statistics values for each variable pair, and p-values. The NP relationships expose even stronger connections.’ (P/L) The correlation between NP and BA is the original pair of the sample, which equals to 0.904, but it increases to an average in the mean. This indicates a very good relationship between the samples, with 0.022 as the standard deviation. A p-value of 0.000 and a t-statistic of 40.658 indicate observable and robust statistical relationships between NP and BA. However, the correlation is not very strong. The correlation between NP and BAb is similar, however it is significant. With a standard deviation of 0.030 and 0.897 sample mean, the original correlation of the samples is 0.900. A statistically significant relationship is evident with a t-statistic of 29.601 and p-values of 0.000, which indicate strong associations between BAb, the Model (BA) and the NP. In particular the relationships between NP and both BAand BAb also show strong links with these variables. These results imply that the management of non-performing loans requires effective oversight and strategic decision-making, which are closely linked to the financial system’s health in this situation.

### The perceived understanding of Board Accountability and Non-performing Loans of Commercial Banks

This section presents the interviewees’ perceived understanding of board accountability and non-performing loans of commercial banks. To obtain a clearer picture, the interviewees were asked to answer each of following questions (. In your opinion, to what extent does the board interfere or help the bank to solve the problem of non-performing 2. How would banks manage non-performing loans? 3. How often do the board sit to discuss issues related to non-performing loans?). and the themes and sub themes generated were presented in the Figure 1 as explained below.

**Figure 1.**
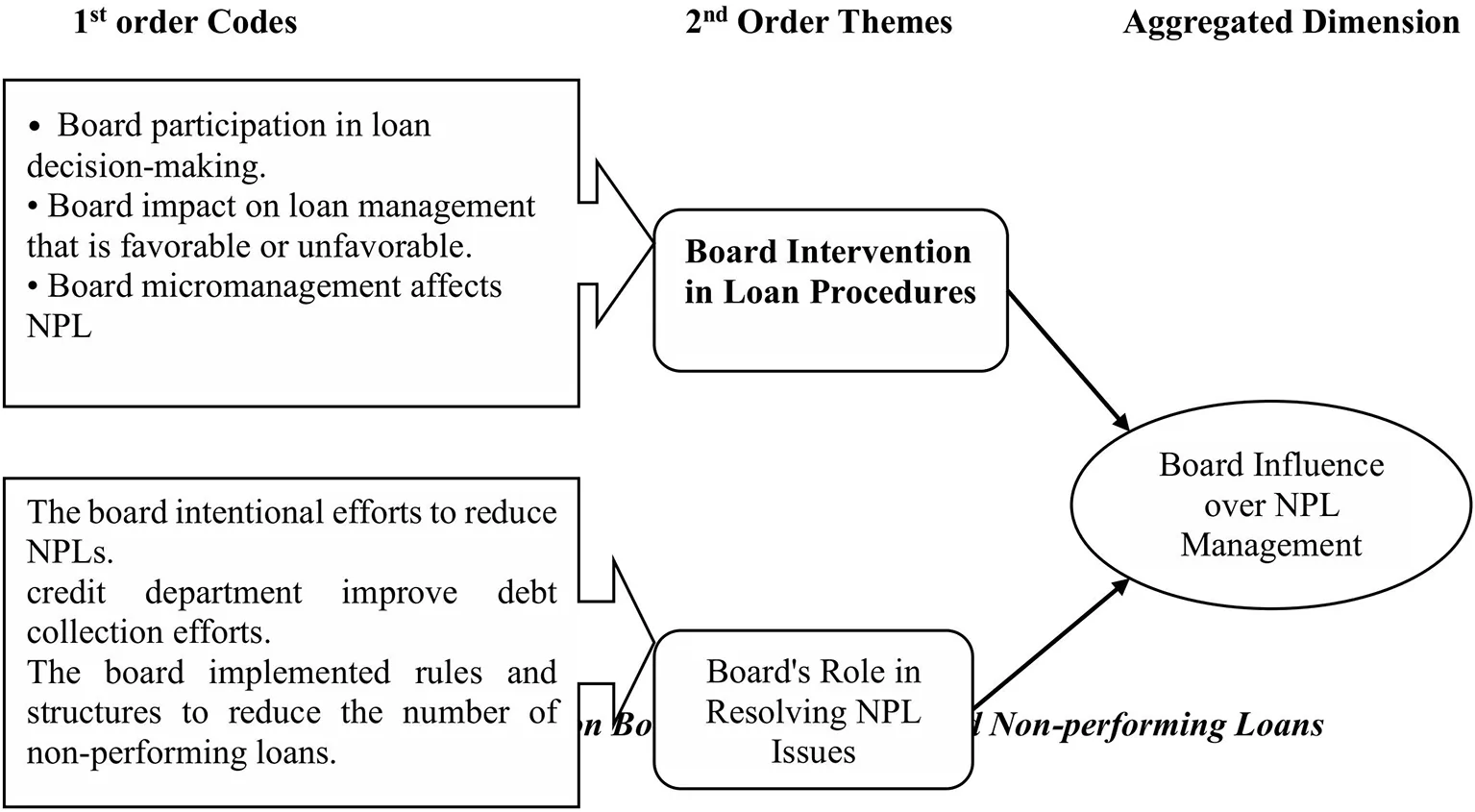
Reality radial diagram on board accountability and non-performing loans.

### Board influence on NPL management

The results in Figure 1 reveal that, generally, interviewees perceived board accountability and non-performing loans of commercial banks as *board influence on non-performing loans management.* On analyzing the transcripts from qualitative interviews, it was established that two major sub themes emerged to mean board influence which are; Extent of Board Interference in Loan Processes and Board’s Role in Solving NPL Issues.

### Board intervention in loan procedures

One of the main themes that emerged in Figure 1 was the extent of board interference in loan processes, with interviewees stating that in most cases, members of the board want to intervene in the loan-giving process by influencing who should get the loans, particularly to favor themselves, their friends, and relatives, which has had an impact on the level of non-performing loans. Interviewees also stated that there is a need to evaluate the nature and frequency of board engagement in loan decision-making, situations of positive vs. negative board influence on loan management, and ways to reduce the impact of board micromanagement on NPL ratios.

### Board’s role in solving NPL issues

After analyzing the transcripts as presented in Figure 1, it was determined that the board’s involvement in solving NPL concerns as a sub-theme, may be brought about by strategic interventions by the board to minimize NPLs. Support for the credit department in enhancing loan recovery operations, as well as the board’s implementation of policies and frameworks to reduce NPL growth, which improves commercial banks’ performance in battling non-performing loans. Interviewees said that it is vital to establish the responsibilities and tasks of the board in commercial banks’ operations, especially when dealing with loan issues, in order to minimize conflict between the board members and management.

## Discussion

Board accountability plays a significant role in the decision-making process of NPLs in commercial banks, as suggested by the findings. The outcomes coincide with earlier research, such as Ahmed et al. (2021) and Balagobei (2019), which found that corporate governance factors, including board accountability, are essential in managing NPLs. This study also supports the argument made by Tarchouna et al. (2022), which suggests that effective governance frameworks play a crucial role in reducing NPLs.

## Conclusion

It is suggested by this study that a well-functioning, accountable board is crucial for mitigating NPLs in commercial banks. Banks must ensure board members are of high moral character and establish clear guidelines to prevent any unauthorized interference with loan procedures, as per the results. The board needs to provide active backing to the credit department for enhancing loan recovery initiatives and establish policies to limit NPL expansion.

## Recommendations

The study recommends that ethical governance and high integrity are crucial when it comes to selecting board members in commercial banks. Policy implementation: Banks must devise clear policies to reduce board interference in loan processes and promote decision-making transparency.

## Ethical approval and consent to participate

The procedures and ethical standards employed in this research were rigorously aligned with the Declaration of Helsinki. The study protocol was reviewed and granted approval by Kampala International University Ethics Committee (Approval Number: KIU-2024-356) and Uganda National Councial for Science and Technology (Approval Number: SS3114ES). Prior to their participation, all subjects were provided with a detailed information sheet explaining the study’s purpose, procedures, potential risks, and benefits. Written informed consent was subsequently obtained from each participant. The confidentiality and anonymity of all participant data were maintained throughout the research.

## Consent to publish declaration

We, the authors, agree to publish this work in F1000 Research and confirm that it is original, unpublished, and not submitted elsewhere. Any personal data included has been approved by those involved, with consent records available if needed.

## Data Availability

Repository name: Zenodo: Mitigating Credit Risk through Corporate Governance: An Investigation into the Causal Pathways from Board Accountability to Reduced Non-Performing Loans in Uganda’s Commercial Banks. data associated with this article can be accessed on https://doi.org/10.5281/zenodo.17249817. This project contains the following underlying data:
•
PhD data set 2025 II.sav (raw survey data collected from employees, board members and clients of selected commercial banks in western Uganda). Repository name: Zenodo: Mitigating Credit Risk through Corporate Governance: An Investigation into the Causal Pathways from Board Accountability to Reduced Non-Performing Loans in Uganda’s Commercial Banks. Extended data associated with this article can be accessed on https://doi.org/10.5281/zenodo.17249817 ( Muniru, 2025). This project contains the following extended data:
• DATA COLLECTION TOOLS FINAL.pdf (full survey instrument used to collect data from participants). •
INFORMED CONSENT APPROVED BY REC.pdf (approved consent form by Kampala International University Research Ethics Committee). Data are available under the terms of the Creative Commons Zero “No rights reserved” data waiver (CC0 1.0 Public domain dedication).
