# Supplementary material for: Mitigating Credit Risk through Corporate Governance: An Investigation into the Causal Pathways from Board Accountability to Reduced Non-Performing Loans in Uganda’s  Commercial Banks
Source: F1000Res. 2025 Oct 14;14:1113. [Version 1] doi: 10.12688/f1000research.171389.1 (PMC13687740; doi:10.12688/f1000research.171389.1)
Supplement: Peer review history

# Reviewer report: Reviewer response for version 1

**DOI:** 10.5256/f1000research.188990.r436537

**Published:** 2026-10-21

**Authors:** Uttam Golder

**Affiliations:** 1 Jashore University of Science and Technology, Jessore District, Khulna Division, Bangladesh

The manuscript addresses a relevant governance issue, but substantial inconsistencies in measurement, statistical reporting, and interpretation undermine its conclusions.

 Clarify the outcome and effect direction. The positive correlation (\(r=0.779\)) is interpreted as reduced NPLs. This is justified only if higher outcome scores represent better NPL management rather than higher NPL levels. Provide the items, coding, and scoring procedure, and distinguish perceived management effectiveness from actual bank NPL ratios.

 Correct statistical interpretations and reconcile results. \(R^2=0.607\) means 60.7% of outcome variance is explained; it does not mean NPLs decline by 60.7%. Adjusted \(R^2\) is not a unit-change effect. Reconcile the reported regression coefficients with the single-predictor model and remove unexplained references to financial disclosure. Table 5 is referenced but not presented.

 Remove unsupported causal claims. Cross-sectional correlations and simple regression cannot establish “causal pathways.” Address omitted variables, reverse causality, and common method bias, and revise the title and conclusions to reflect associations.

 Resolve sampling and independence concerns. Reconcile 232 sampled participants, 195 reported responses, and 189 analysed observations. Explain recruitment, exclusions, respondent categories, and collection dates. Respondents are nested within only three banks; employee counts cannot substitute for independent bank-level observations when drawing conclusions about governance and NPLs.

 Strengthen the qualitative component and contribution. Explain interview selection, coding, researcher involvement, and evidence sufficiency; include anonymized quotations and integrate findings with the quantitative results. Update the literature, substantiate novelty claims, verify references, and acknowledge the limited geographical and institutional coverage.

**Suggested literature:** The authors may consider these studies where relevant:

 Refer (1)—directly relevant to governance–NPL relationships and endogeneity.

 Refer (2)—useful for distinguishing credit-risk indicators from bank-performance outcomes.

 Refer (3)—a broader contextual reference if bank financial structure is discussed; its relevance is less direct.

Is the work clearly and accurately presented and does it cite the current literature?

Partly

If applicable, is the statistical analysis and its interpretation appropriate?

Yes

Are all the source data underlying the results available to ensure full reproducibility?

Yes

Is the study design appropriate and is the work technically sound?

Partly

Are the conclusions drawn adequately supported by the results?

Yes

Are sufficient details of methods and analysis provided to allow replication by others?

Yes

Reviewer Expertise:

FinTech, Bank

I confirm that I have read this submission and believe that I have an appropriate level of expertise to confirm that it is of an acceptable scientific standard, however I have significant reservations, as outlined above.

## References

[1] Hossain et al. (2025), Effectiveness of Internal Corporate Governance Mechanisms in Controlling Non-Performing Loans of an Emerging Economy.

[2] Hossain and Golder (2022), The Impact of Credit Risk Management on the Financial Performance of the Banks Listed on the Dhaka Stock Exchange.

[3] Shahriar et al. (2021), Factors Shaping Capital Structure: Evidence from Private Commercial Banks in Bangladesh.

# Reviewer report: Reviewer response for version 1

**DOI:** 10.5256/f1000research.188990.r436536

**Published:** 2026-01-06

**Authors:** Nurna Aziza

**Affiliations:** 1 Universitas Bengkulu, Bengkulu, Indonesia

**Strength**

 This research is very interesting and adds to the literature on the condition of the commercial bank industry in Western Uganda, which has experienced many bankruptcies due to credit risk, and adds knowledge related to strategies for addressing the issue of credit failure/credit risk.

 This research has the strength of using two data sources (mixed methods), namely quantitative data and qualitative data through interviews analyzed with NVivo software. These data sources strengthen the research results to meet the research gaps that the researcher wants. The results of the interviews obtained information on the level of the board's interference in loan transactions and the board's involvement in managing problems in NPLs. The results of this study have informed that there is a council intervention in the loan or credit process resulting in higher NPLs. This is the problem that many banks experience credit risk, and there is a need for board accountability, not just about the existence of the board. In this study, the results of the correlation value testing of 60% showed that board members are responsible by doing their duties by following the guiding principles and policies, so that commercial banks' non-performing loans are reduced and vice versa.

**Disadvantages**

1. This study has not explained the period of data obtained.
2. The results of the interview data sources have not been explored properly, even though this is an additional value of this study.
3. It is better to present the results of the correlation relationship (SEM PLS results) so that it is clearer. Even though there is already a link to the data source.

Is the work clearly and accurately presented and does it cite the current literature?

Yes

If applicable, is the statistical analysis and its interpretation appropriate?

Yes

Are all the source data underlying the results available to ensure full reproducibility?

Partly

Is the study design appropriate and is the work technically sound?

Partly

Are the conclusions drawn adequately supported by the results?

Yes

Are sufficient details of methods and analysis provided to allow replication by others?

Yes

Reviewer Expertise:

Financial accounting, environmental accounting, sustainability reporting

I confirm that I have read this submission and believe that I have an appropriate level of expertise to confirm that it is of an acceptable scientific standard.

# Reviewer report: Reviewer response for version 1

**DOI:** 10.5256/f1000research.188990.r424762

**Published:** 2025-10-28

**Authors:** Ahmad Nurkhin

**Affiliations:** 1 Universitas Negeri Semarang, Semarang, Central Java, Indonesia

This is an interesting article that applies mixed methods to investigate the impact of GCG mechanisms on banking non-performing loans (NPLs) in Western Uganda. However, the qualitative research is less prominent.

 The author could have explored further how the design and type of qualitative research were determined (explanatory, exploratory, or other), the data obtained from six participants through structured interviews, and how the results were analyzed. The qualitative description and discussion of the results are less robust than the quantitative presentation.

Is the work clearly and accurately presented and does it cite the current literature?

Yes

If applicable, is the statistical analysis and its interpretation appropriate?

Yes

Are all the source data underlying the results available to ensure full reproducibility?

Yes

Is the study design appropriate and is the work technically sound?

Partly

Are the conclusions drawn adequately supported by the results?

Yes

Are sufficient details of methods and analysis provided to allow replication by others?

Yes

Reviewer Expertise:

accounting and education.

I confirm that I have read this submission and believe that I have an appropriate level of expertise to confirm that it is of an acceptable scientific standard, however I have significant reservations, as outlined above.

# Response

**Published:** 2025-10-28

**Authors:** Muniru Sewanyina

**Affiliations:** Business Administration, Kampala International University - Western Campus, Bushenyi, Western Region, Uganda

Thank you for your generous observation. The study used a mixed methods design; however, it was more quantitative, that is why the qualitative part was given less attention
